# A Rare Presentation of an Ocular Ischemic Syndrome Complicated by Neovascular Glaucoma in a Patient With Mild Carotid Artery Stenosis: A Case Report

**DOI:** 10.7759/cureus.83336

**Published:** 2025-05-02

**Authors:** Muhamad Zulhilmi Akmal Zainuddin, Jemaima Che Hamzah, Teck Chee Cheng

**Affiliations:** 1 Department of Ophthalmology, Faculty of Medicine, Universiti Kebangsaan Malaysia, Kuala Lumpur, MYS

**Keywords:** atherosclerosis, carotid artery stenosis, internal carotid artery, neovascular glaucoma, ocular ischemic syndrome, ophthalmic artery, rubeosis

## Abstract

A 72-year-old male with hypertension, dyslipidemia, ischemic heart disease, chronic kidney disease, and gout presented with painless blurred vision in the right eye, with intermittent ipsilateral headache for six months. Upon presentation, the right eye vision was 6/60 pinhole 6/36, with the presence of a relative afferent pupillary defect. An anterior segment examination revealed right eye ciliary injection and corneal edema, with anterior chamber cells and fibrin. The right eye intraocular pressure (IOP) was 52 mmHg, and gonioscopy showed an open angle with rubeosis at the angle. The right eye fundus revealed a glaucomatous optic disc with a 0.7 cup-to-disc ratio, multiple dot-blot hemorrhages over the mid-peripheral retina, and narrowed retinal arteries suggesting ocular ischemic syndrome (OIS). The left eye examination was unremarkable. The diagnosis was further confirmed by fundus fluorescein angiography, which showed a marked delay in arterio-venous transit time with a profound area of capillary fall-out at the peripheral retina. To tackle the ischemic component, a pan-retina photocoagulation laser was conducted. Topical and systemic antiglaucoma medications, topical steroids, and cycloplegics were commenced. He subsequently required a glaucoma drainage device in his right eye to control the IOP. He was investigated for the cause of OIS. Ultrasound carotid Doppler and computed tomography angiography of carotid arteries revealed bilateral internal carotid artery atherosclerotic disease with less than 50% stenosis. Double antiplatelet therapy was commenced to reduce the risk of cerebrovascular and cardiovascular events. Two months after the operation, his right eye's intraocular pressure was controlled without antiglaucoma medications. After seven months of follow-up, his right eye vision improved to 6/12 with an IOP of 10 mmHg.

## Introduction

Neovascular glaucoma (NVG) is a secondary glaucoma that is associated with iris and iridocorneal angle rubeosis, connective tissue growth, and elevated intraocular pressure (IOP) [1,2]. Changes in the optic disc are non-specific, and glaucomatous optic neuropathy is not required for the diagnosis of NVG [2]. NVG is often refractory to treatment, neither medical nor surgical therapies, and hence it has become a sight-threatening condition with high rates of severe impairment of visual acuity, which can be incurable and ultimately lead to blindness [1].

Ninety-five percent (95%) of cases of NVG are due to retinal ischemia [1,2]. The most common etiologies are proliferative diabetic retinopathy (PDR) (33%), ischemic central retinal vein occlusion (CRVO) (33%), and ocular ischemic syndrome (OIS) (13%) [1-4].

OIS is a rare condition in which 7.5 people per million are diagnosed annually [3,5]. It is characterized by ischemia of the anterior and posterior segments of the eye, secondary to chronic ocular hypoperfusion, which is commonly associated with severe carotid artery stenosis or occlusion [2,6]. We reported a case of NVG secondary to OIS with mild carotid artery stenosis based on ultrasound carotid doppler (UCD) and computed tomography angiography (CTA) of carotid arteries.

This article was previously presented as an e-poster at the International Virtual 2023 Medical Research Symposium on the 7th and 8th of December 2023.

## Case presentation

A 72-year-old male, a non-smoker, with underlying hypertension, dyslipidemia, ischemic heart disease, chronic kidney disease, and gout, presented to the Ophthalmology clinic with a six-month history of right eye blurring of vision, which worsened for the past two months. The blurring of vision was described as generalized haziness and progressive. It was associated with intermittent ipsilateral right-sided headache, which was throbbing in nature. However, there was no nausea or vomiting. He denied any eye pain, jaw claudication, or scalp tenderness. There were no floaters or flashes of light. There was no history of ocular trauma. For past ocular history, he had bilateral uneventful cataract surgery with intraocular lens implantation done many years prior.

**Figure 1:**
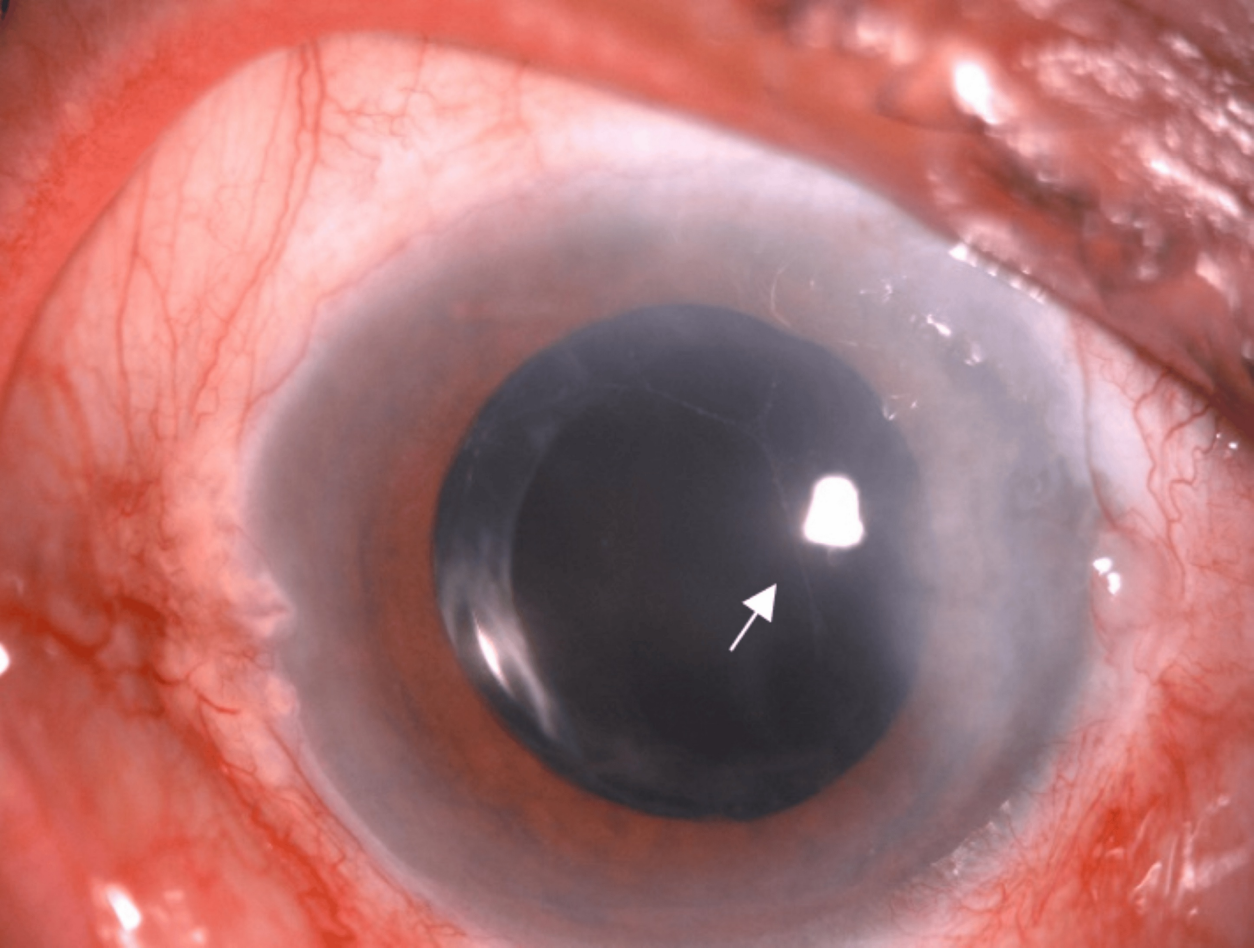
Anterior segment photo of the right eye showing ciliary injection, mild cornea edema, and fibrin (white arrow) in the anterior chamber

**Figure 2:**
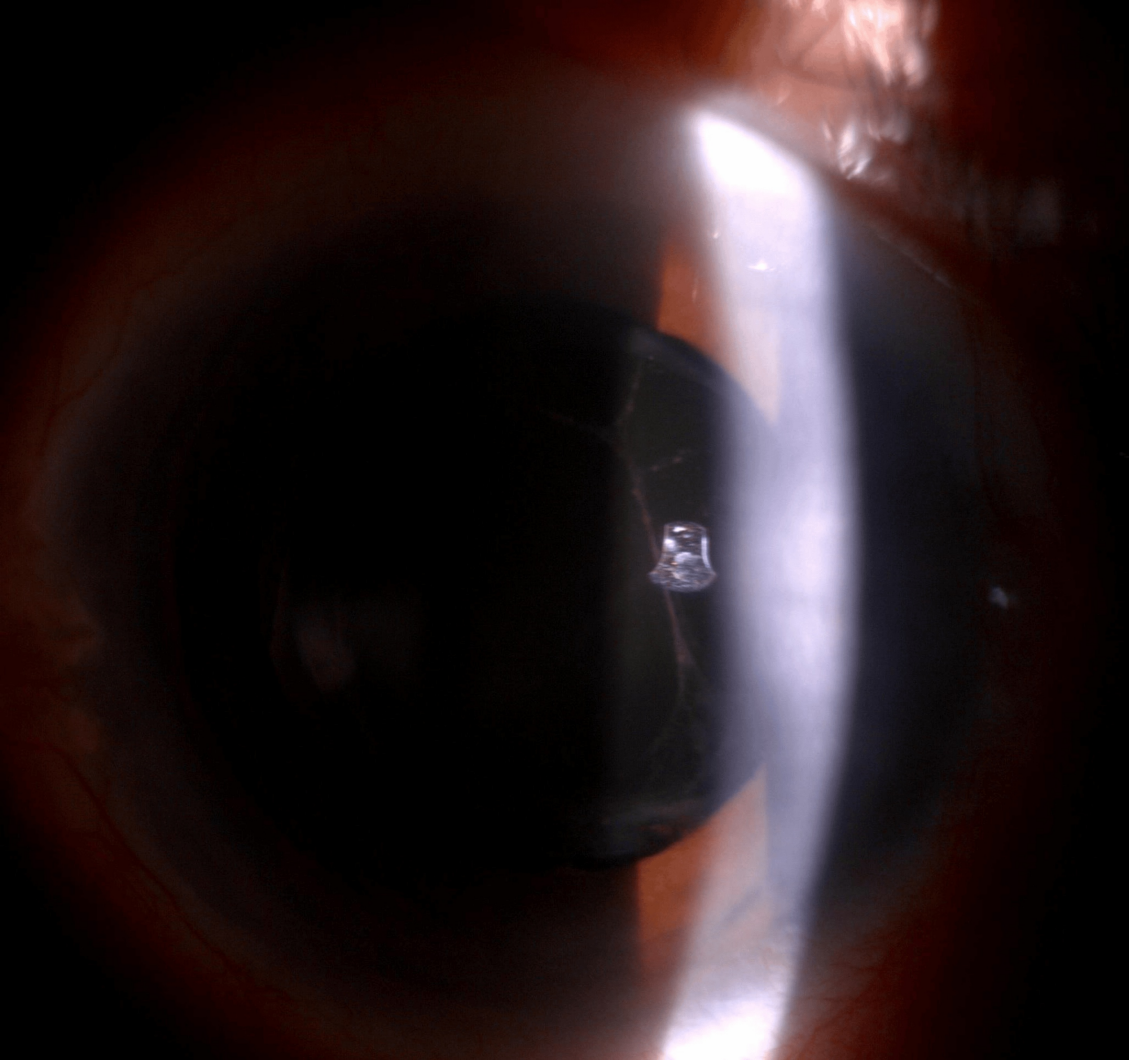
Anterior segment photo with a slit lamp view of the right eye showing mild cornea edema

**Figure 3:**
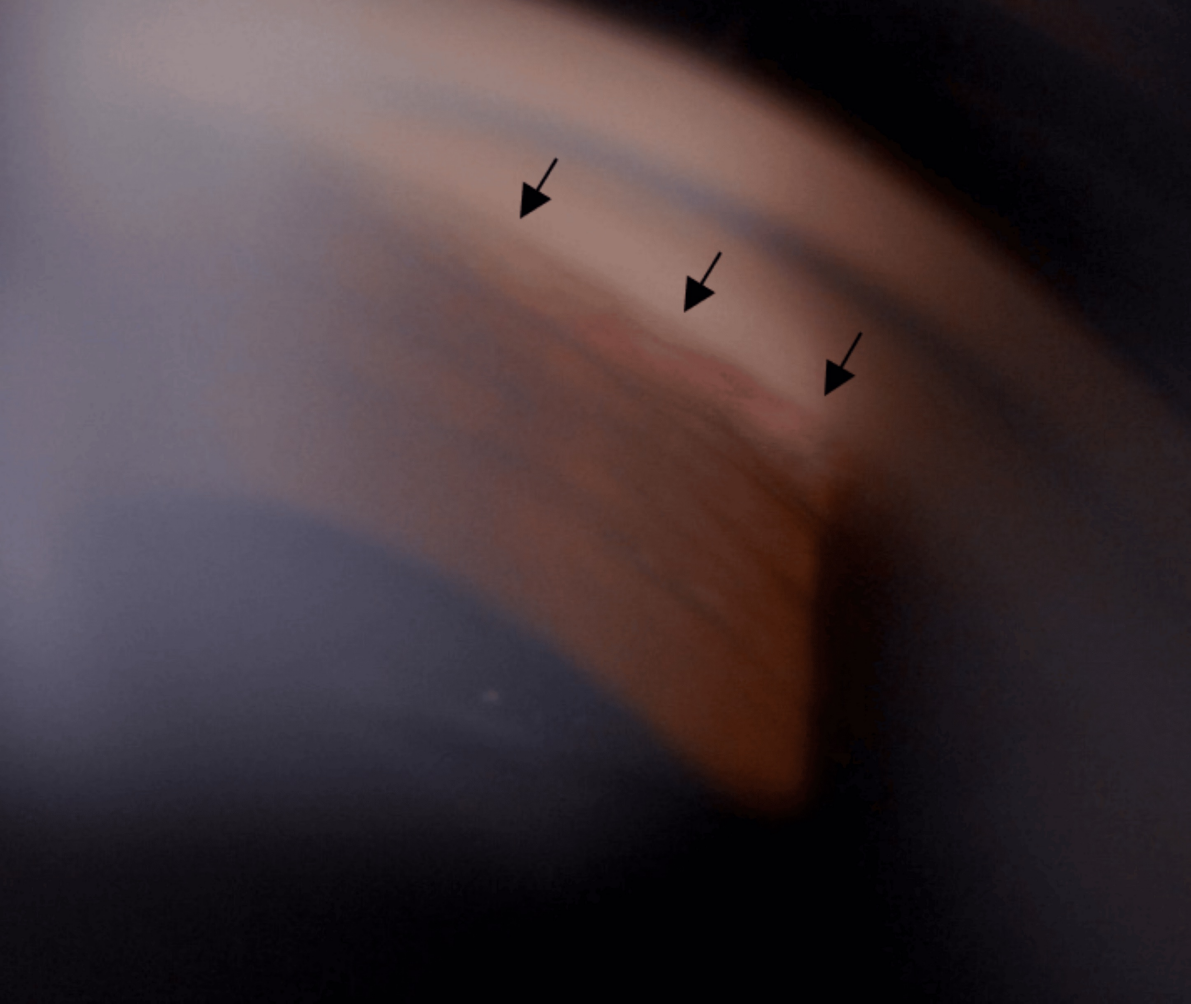
Gonioscopy of the right eye showing rubeosis (black arrows) at the angle

**Figure 4:**
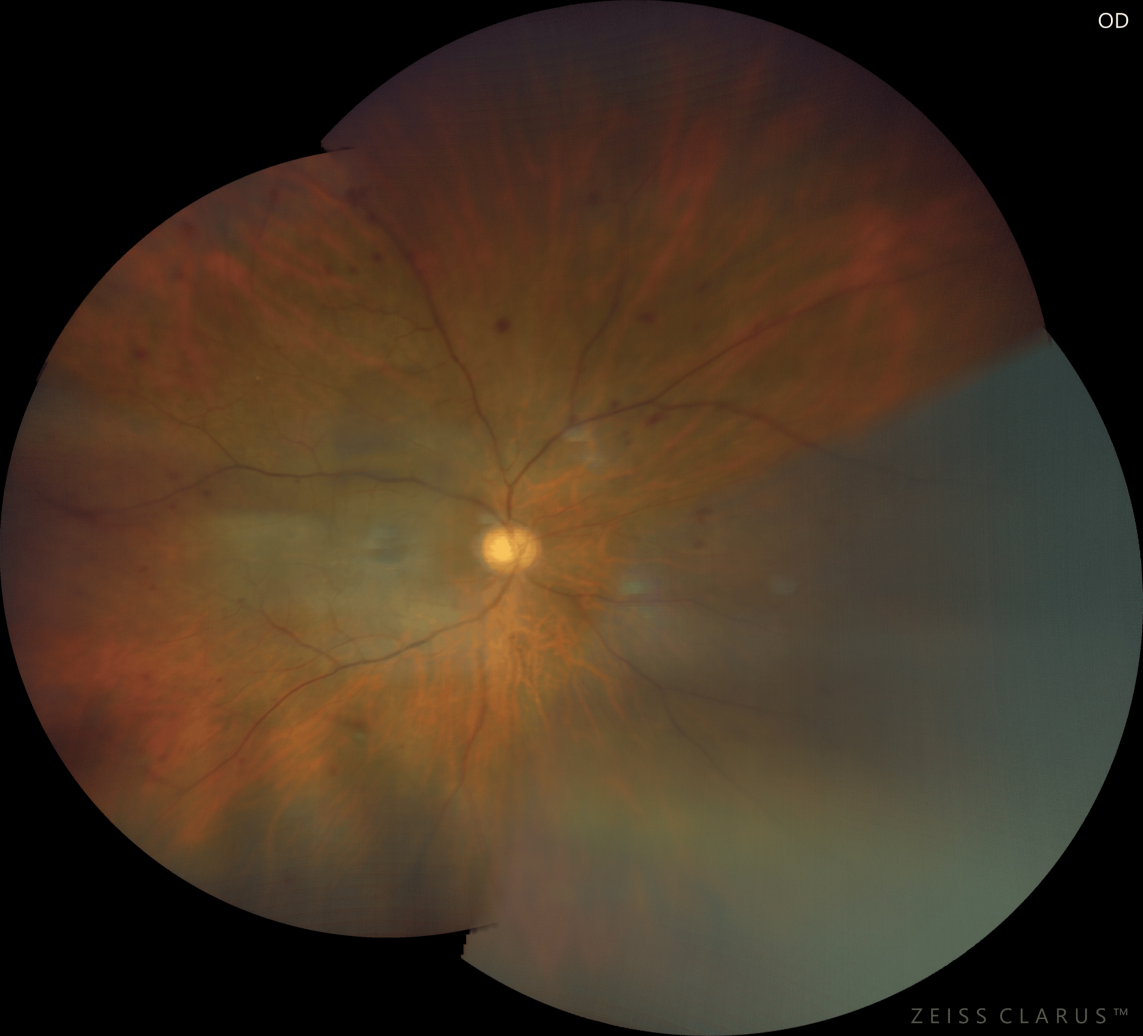
Fundus photo of the right eye showing a glaucomatous optic disc with dot-blot hemorrhages over the mid-peripheral retina and narrowed retinal arteries suggesting ocular ischemic syndrome

During the presentation, visual acuity was 6/60, improving to 6/36 with pinhole correction over the right eye and 6/6 over the left eye. Relative afferent pupillary defect (RAPD) was present over the right eye. Anterior segment examination over the right eye (Figure 1) showed ciliary injection over the conjunctiva with corneal edema (Figure 2). The anterior chamber (AC) was deep, with the presence of cells grading 1+ and fibrin. Intraocular pressure (IOP) was 52 mmHg. Gonioscopy revealed an open angle with the presence of rubeosis at an angle of almost 360 degrees (Figure 3). The intraocular lens (IOL) was stable with no posterior capsule opacification. The right eye fundus (Figure 4) showed a glaucomatous optic disc with a cup-to-disc (CDR) ratio of 0.7 and a normal macula. There were multiple dot-blot hemorrhages over the mid-peripheral retina and narrowed retinal arteries; however, no neovascularization was seen. These findings were suggestive of an ocular ischemic syndrome (OIS). On the other hand, the left eye anterior segment examination was unremarkable, with normal IOP. The left eye fundus revealed a pink optic disc with a CDR of 0.3 and no retinal hemorrhages.

**Figure 5:**
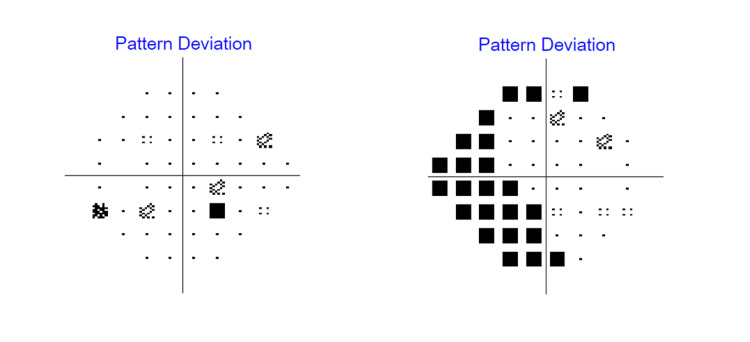
The Humphrey visual field (HVF) 24-2 perimetry test upon initial presentation showing right eye superonasal and inferonasal scotoma

A Humphrey visual field perimetry test (Figure 5) was performed to assess the severity of glaucomatous damage to the patient. There was the presence of superonasal and inferonasal scotoma over the right eye upon presentation.

**Figure 6:**
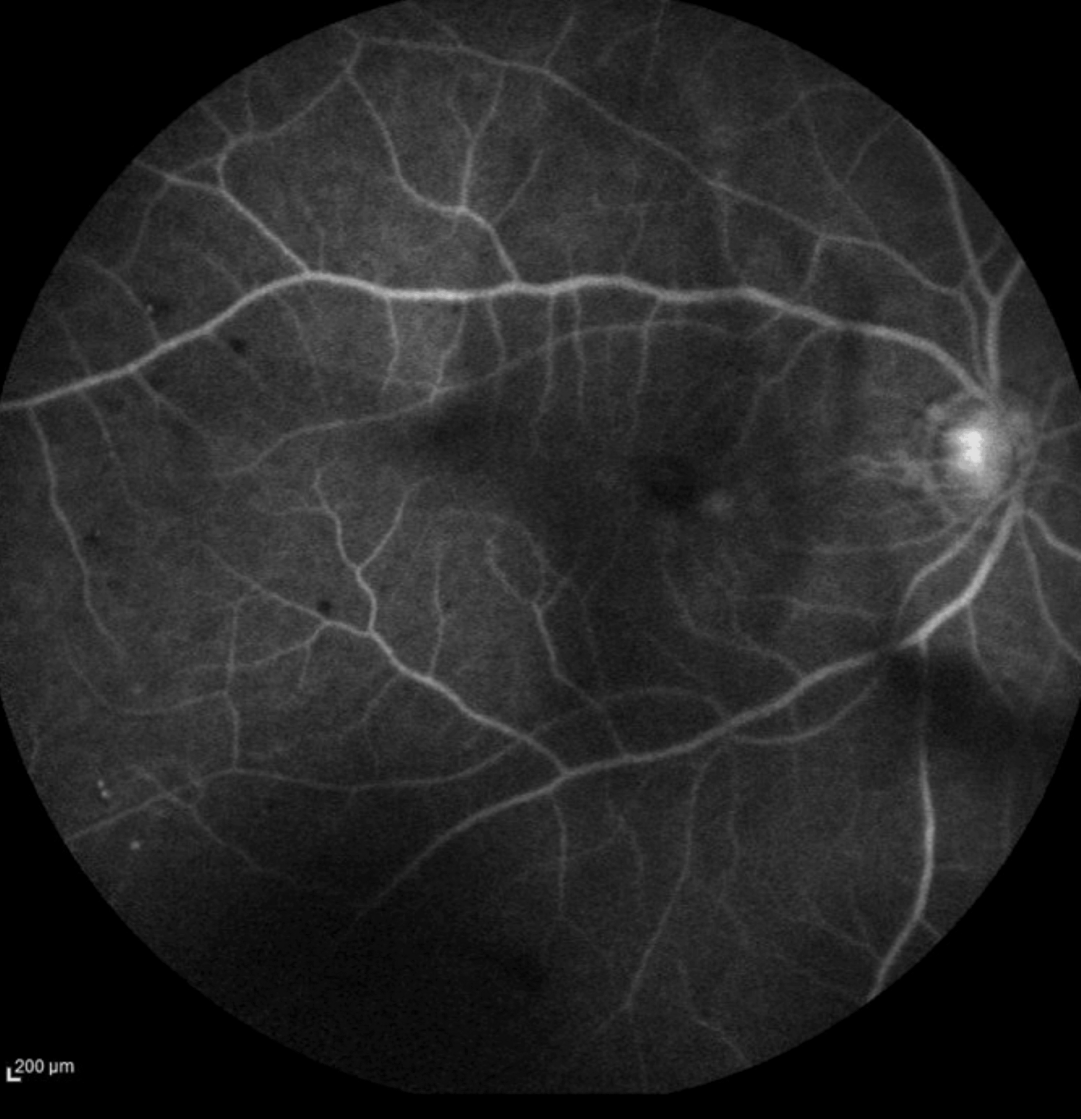
Fundus fluorescein angiography of the right eye at a late recirculation stage showing profound areas of capillary fallout at the peripheral retina

The diagnosis of OIS was further confirmed with fundus fluorescein angiography (FFA), which showed a marked delay in arterio-venous transit time (20 seconds), with profound areas of capillary fall-out at the peripheral retina (Figure 6).

The patient was then investigated for the cause of OIS. Full blood count (including hemoglobin, platelets, and hematocrit), erythrocyte sedimentation rate, fasting blood glucose, and lipid levels were within normal range. Ultrasound carotid Doppler (UCD) was done and revealed bilateral internal carotid artery (ICA) atherosclerotic disease by calcified plaques at bilateral carotid bulbs with 36% and 47% luminal narrowing on the right and left, respectively. Computed tomography angiography (CTA) of the carotid artery further confirmed the atherosclerotic changes at the cavernous segment and supraclinoid segment of bilateral ICA with approximately 25% stenosis.

**Figure 7:**
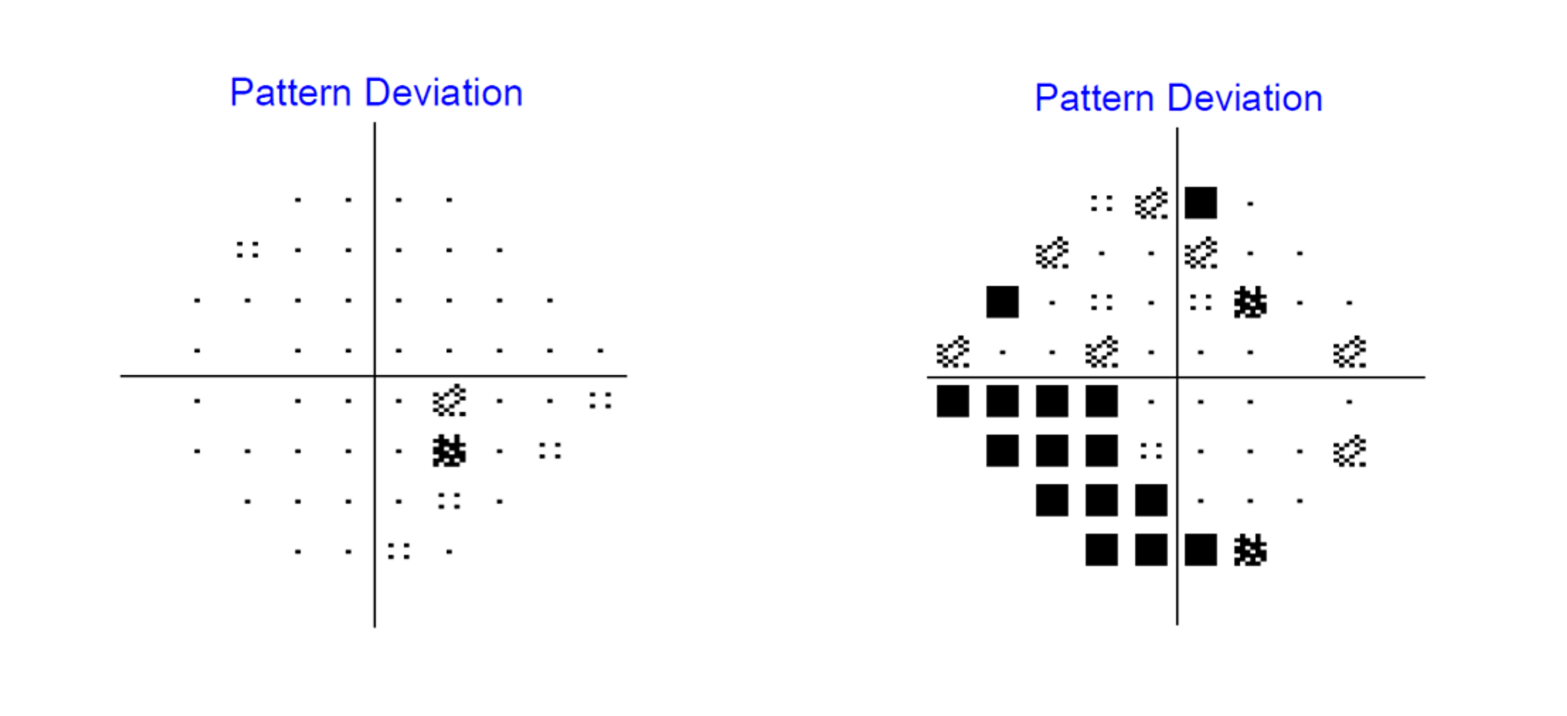
The Humphrey visual field (HVF) 24-2 perimetry test after seven months of follow-up showing persistent inferonasal scotoma in the right eye

To tackle the ischemic component of OIS, a panretinal photocoagulation (PRP) laser was carried out. Topical and systemic antiglaucoma medications were commenced to control the high IOP, together with topical steroid and cycloplegic to control the ocular inflammation and prevent the formation of synechiae, respectively. He subsequently underwent an Ahmed ClearPath 250 implant (New World Medical, Inc., Rancho Cucamonga, CA, US) in his right eye to further control the IOP. He was also started on double antiplatelet therapy to reduce the risk of cerebrovascular and cardiovascular events, given the presence of symptomatic carotid artery stenosis. Two months after the Ahmed ClearPath 250 implant operation, his right eye IOP was controlled without any topical or systemic antiglaucoma medication. After seven months of follow-up, his right eye vision improved to 6/12 with a stable IOP of 10 mmHg. Right eye rubeosis regressed, and the anterior chamber was quiet with no more cells or fibrin. The topical steroid was tapered off. However, on fundus examination, the optic disc was pale with a CDR of 0.7. The Humphrey visual field perimetry test was performed and revealed a persistent inferonasal scotoma over the right eye, which indicates permanent glaucomatous damage (Figure 7).

## Discussion

OIS is a disease characterized by anterior and posterior segment ischemia secondary to chronic ocular hypoperfusion [2,6]. OIS occurs when there is a blockage of the ophthalmic artery, which is a branch of the internal carotid artery (ICA). The visual prognosis is poor in OIS, with 58% of corrected visual acuity below counting fingers at one year [3]. Besides that, the mortality rate within 5 years of OIS onset is 40%, with cardiovascular disease as the leading cause of death, followed by stroke [3,6].

The major cause of OIS is the presence of atheromatous plaque in the carotid artery, which is also known as carotid artery atherosclerosis [2,5]. The atheromatous plaque in the carotid artery will lead to continuous ocular ischemia, which will make the eye more susceptible to OIS. The risk factors include elderly patients typically between 50 to 60 years old with underlying systemic diseases such as diabetes mellitus, hypertension, and dyslipidemia [7]. Other causes that can contribute to OIS include dissecting aneurysm of the carotid artery, Takayasu arteritis, aortic arch aneurysm, Behcet’s disease, trauma or inflammation causing stenosis of carotid arteries, and complications after intravitreal anti-vascular endothelial growth factor (anti-VEGF) injections and post radiotherapy for nasopharyngeal carcinoma [6].

Carotid artery stenosis is a common condition, and it is related to the development of atherosclerotic plaque in the ICA, which has an extracranial (in the neck) and intracranial (within the skull) course [8]. Atherosclerosis is a chronic arterial disease that involves the accumulation of cholesterol-lipid-calcium deposits in the arterial walls. According to the North American Symptomatic Carotid Endarterectomy Trial (NASCET) classification, carotid artery stenosis can be divided into four categories, which are mild/low (less than 50% stenosis), moderate (50-69% stenosis), severe (70-99% stenosis), and total occlusion (100%) [9-11]. Our patient demonstrated bilateral internal carotid artery (ICA) atherosclerotic disease with 25% stenosis, which falls into a mild category.

Typically, a stenosis of 90% or more of the common carotid artery or ICA is found, leading to decreased ocular perfusion [5,6,12]. Kim et al. found that 50% of the patients with OIS had a complete occlusion of the carotid artery on the affected side, and among 10% of them, bilateral complete carotid artery occlusion was found [5]. Nonetheless, in cases of complete carotid artery occlusion, OIS may not occur if collateral circulation is well-developed [5,6]. Terelak-Borys et al. reported that OIS is still possible in a stenosis of 50% in the absence of collateral circulation [6]. Our patient developed unilateral OIS with bilateral carotid artery stenosis of less than 25% of bilateral carotid artery stenosis, which was later complicated by neovascular glaucoma (NVG).

Patients with OIS can have various presentations, the most common being decreased visual acuity of the affected eye in 90% of patients. It is usually due to acute or chronic retinal ischemia, or damage to the optic nerve due to secondary glaucoma such as NVG. Patients can also present with sudden visual loss, with a cherry-red spot observed on fundus examination, signifying central retinal artery occlusion as one of the complications of OIS [6]. Ocular or periorbital pain is another common presentation for patients with OIS, which is about 40%. It can be secondary to NVG with raised IOP or caused by hypoxia of the eyeball and/or dura mater. Our patient presented with chronic blurring of vision and intermittent ipsilateral headache. He did not complain of ocular pain, most likely due to the gradual rise in his IOP.

Sixty-six percent (66%) of patients with OIS were found to have neovascularization of the iris and iridocorneal angle, which resulted in impairment of the aqueous humor outflow, leading to an increase in IOP and development of NVG [6]. Rubeosis at the angle can also lead to synechial angle closure if left untreated. However, ocular hypotony may still be possible despite having fibrovascular tissue and synechial angle closure. This can be explained by ischemia of the ciliary body secondary to hypoperfusion, which leads to reduced aqueous humor production [2,6]. Other anterior segment signs of OIS may include dilatation of conjunctival and episcleral vessels, and corneal edema [6].

Posterior segment signs are more frequent than anterior segment signs. Most of the time, the retinal arteries appear narrowed; meanwhile, the retinal veins are dilated. Retinal hemorrhages are very characteristic of OIS, and they are seen in about 80% of affected eyes [6]. The retinal hemorrhages are not numerous and are mostly located in the external retinal layers and at the mid-periphery. They are most probably due to leakage from the small retinal vessels or from the ruptured capillary aneurysms. Macula edema can also occur in OIS, and it is due to diffuse macular capillary telangiectasia together with a capillary microaneurysm. As a result of increased production of vascular endothelial growth factor (VEGF), neovascularization can occur at the optic disc and retina, but more often at the optic disc.

Imaging of the carotid artery is crucial in patients with OIS to treat the underlying cause. It can be further divided into non-invasive and invasive tests. Non-invasive imaging, including UCD and ocular plethysmography, allows the detection of carotid artery stenosis in at least 75% of cases. On the other hand, invasive tests like carotid arteriography, CTA, and magnetic resonance angiography (MRA) can be performed in advanced cases before surgery is planned for carotid artery stenosis [6,7]. Besides that, ocular tests, such as FFA and indocyanine green angiography (ICGA), can also be used to establish the diagnosis of OIS. A prolonged circulation time of the arm to the choroid and the arm to the retina is a frequent and specific sign of OIS in FFA [6]. Irregular and/or prolonged retinal filling time is the most specific FFA sign of OIS; however, it is not sensitive, as it is only found in 60% of patients with OIS. The most sensitive angiographic sign of OIS is prolonged retinal arteriovenous time, which is present in up to 95% of cases. However, it is not specific to OIS. Abnormalities of the choroidal circulation can be seen in ICGA with a prolonged arm-to-choroid and intrachoroidal circulation time [6]. Choroidal hypoperfusion can be detected in areas of vascular filling defects in the posterior pole.

NVG is one of the sight-threatening complications of OIS and is usually refractory to medical treatment [3,6]. PRP laser is mandatory to reduce retinal oxygen demand by ablation of the peripheral retina, which aims to inhibit neovascularization, especially at the iridocorneal angle. However, it is only effective in 36% of cases, as choroidal ischemia alone is sufficient to induce neovascularization without retinal ischemia [6,12]. In the case where the fundus is not visible due to media opacities or poor pupil dilatation, alternative methods, such as transconjunctival cryotherapy in the mid-peripheral and peripheral retina and trans-scleral diode laser retinopexy, can be considered [3,12].

When NVG is refractory to medical therapy, filtering surgery is indicated. However, due to the risk of intra- and postoperative complications and the low success rate of trabeculectomy in NVG, glaucoma drainage device implantation is a preferred surgical option. Our patient did not respond to maximal medical therapy and subsequently received an Ahmed ClearPath implant to control his IOP.

For severe carotid artery stenosis, carotid artery endarterectomy (CEA) or carotid artery stenting (CAS) should be considered [3,6]. It is effective in symptomatic carotid artery stenosis of 70-90% and in asymptomatic stenosis of at least 60%. However, in cases of complete occlusion, arterial bypass surgery should be performed [6]. CEA is performed to remove the plaque from inside the carotid artery, and it is known to reduce the risk of stroke in symptomatic patients. It has also been proven to increase the blood flow in ocular arteries and prevent ischemic changes [3]. CAS, on the other hand, is a procedure where an expanding stent is inserted into the carotid artery to increase the blood flow to the area blocked by plaque. CAS is indicated in patients who have had previous radiation, neck surgery, recurrent stenosis, tracheostomy, or complicated surgery with stenosis above the second cervical (C2) vertebral level and high-risk patients, such as patients with unstable angina, congestive cardiac failure, and recent myocardial infarction [3]. Another option is extracranial-intracranial (EC-IC) arterial bypass surgery, which creates an anastomosis between the extracranial branch of the superficial temporal artery and the intracranial branch of the tunica media artery. It is indicated when the common carotid artery or internal carotid artery is completely occluded or when there is difficulty assessing at the C2 or higher level due to stenosis [3]. Apart from vascular surgical treatment, antiplatelet medical therapy is essential in carotid artery stenosis management [8]. Antiplatelets help to reduce the risk of further neurological events from unstable plaque. In symptomatic carotid artery stenosis, the use of aspirin, clopidogrel, aspirin-dipyridamole, or ticagrelor is a standard treatment [13]. Our patient was referred to the Neurology and Vascular team, where double antiplatelet treatment was given in view of the mild carotid artery stenosis.

## Conclusions

The occurrence of OIS in carotid artery stenosis seems independent of the degree of stenosis. Commonly, it is associated with severe carotid artery stenosis or occlusion; however, it can still occur in mild carotid artery stenosis. Prompt diagnosis and multidisciplinary management are important to prevent sight-threatening and life-threatening conditions such as neovascular glaucoma and cerebrovascular accidents.
